# Lactic Acid Bacteria-Derived Antimicrobial and Anti-Biofilm Strategies: Mechanisms, Functional Molecules, and Emerging Biomaterial Applications

**DOI:** 10.3390/ijms27135749

**Published:** 2026-06-25

**Authors:** Weichen Gong, Harum Fadhilatunnur, Miaya Kanazawa, Julio Villena, Keita Nishiyama, Haruki Kitazawa

**Affiliations:** 1 Laboratory of Animal Food Function, Graduate School of Agricultural Science, Tohoku University, Sendai 980-8572, Japan; 2 Livestock Immunology Unit, International Education and Research Center for Food and Agricultural Immunology (CFAI), Graduate School of Agricultural Science, Tohoku University, Sendai 980-8572, Japan; 3 Department of Food Science and Technology, Faculty of Agricultural Technology, IPB University, Bogor 16680, Indonesia; 4 Laboratory of Respiratory Immunology (LaRI), Division of Animal Immunology and Omics, International Education and Research Center for Food and Agricultural Immunology (CFAI), Graduate School of Agricultural Science, Tohoku University, Sendai 980-8572, Japan

**Keywords:** lactic acid bacteria, antimicrobial agent, anti-biofilm

## Abstract

Lactic acid bacteria (LAB), particularly members of the genus *Lactobacillus*, have emerged as promising biological agents with antimicrobial and anti-biofilm properties. While numerous individual studies have reported their inhibitory effects against pathogenic microorganisms, a systematic understanding that integrates their functional components, molecular mechanisms, and material-based applications remains lacking. In this review, we provide a comprehensive and component-oriented overview of *LAB*-mediated antimicrobial strategies. We first summarize secreted factors, including organic acids, bacteriocins, hydrogen peroxide, and extracellular vesicles, which collectively contribute to direct pathogen inhibition and environmental modulation. We then discuss cell-associated components such as surface-layer proteins and exopolysaccharides, highlighting their roles in adhesion interference and competitive exclusion. In addition, we examine whole-cell effects, including niche competition, quorum sensing disruption, and host immune modulation. Importantly, we place particular emphasis on the anti-biofilm activity of lactobacilli, detailing mechanisms involved in the prevention of the pathogen initial adhesion, disruption of extracellular polymeric substance matrices, and destabilization of mature biofilms. Finally, we explore emerging strategies that integrate lactobacilli with biomaterials, particularly hydrogel-based systems, to achieve controlled delivery, enhanced stability, and sustained antimicrobial activity. These biohybrid approaches represent a promising direction for the development of next-generation antimicrobial materials. These findings support the concept of LAB-based living antimicrobial materials as a next-generation strategy to combat biofilm-associated infections. Overall, this review aims to bridge the gap between molecular functions and translational applications of lactobacilli, providing new insights into its potential as a versatile platform for antimicrobial and anti-biofilm interventions.

## 1. Introduction

Biofilm-associated infections represent a major and persistent challenge in both clinical and industrial settings. Microbial biofilms, characterized by structured communities of microorganisms, exhibit markedly increased resistance to antibiotics, host immune responses, and environmental stresses [1,2]. These properties contribute to the chronicity of infections and are closely linked to the global rise in antimicrobial resistance (AMR) [3]. Conventional antimicrobial strategies, which primarily target planktonic bacteria, are often insufficient to eradicate biofilm-associated pathogens, highlighting the urgent need for alternative or complementary approaches. Notably, sub-inhibitory concentrations of certain antibiotics have been reported to paradoxically promote biofilm formation, further complicating treatment outcomes [4,5,6,7,8]. For example, β-lactam antibiotics such as ampicillin and cefotaxime have been shown to enhance biofilm formation in *Escherichia coli* and *Staphylococcus aureus*, while aminoglycosides can stimulate biofilm development in *Pseudomonas aeruginosa* through the induction of stress-response pathways [6,7,8].

In this context, lactic acid bacteria (LAB), particularly members of the lactobacilli group (the former genus *Lactobacillus)*, have gained increasing attention as promising biological agents with intrinsic antimicrobial and anti-biofilm activities [9]. Notably, the former genus Lactobacillus underwent a major taxonomic revision in 2020 and was reclassified into 25 genera, including *Lactiplantibacillus*, *Lacticaseibacillus*, *Limosilactobacillus*, and several others, based on phylogenomic and comparative genomic analyses. Throughout this review, the term “lactobacilli” is used as a collective and informal designation encompassing these phylogenetically related genera for simplicity and readability [10]. Species from the lactobacilli group are widely distributed in diverse ecological niches, including the human gastrointestinal tract, oral cavity, and fermented foods, and are generally recognized as safe and beneficial for hosts [11]. Increasing evidence has demonstrated that lactobacilli can inhibit a broad spectrum of pathogenic microorganisms through multiple mechanisms, including the production of antimicrobial compounds, competition for ecological niches, and modulation of host immune responses [12].

Despite extensive research, current understanding of lactobacilli-mediated antimicrobial activity remains largely fragmented. Many studies focus on individual strains or isolated factors, such as organic acids or bacteriocins, without integrating these findings into a unified framework that connects molecular components with their underlying mechanisms and functional outcomes. Moreover, while the anti-biofilm potential of lactobacilli have been increasingly recognized, mechanistic insights into how different bacterial components contribute to distinct stages of biofilm formation and disruption remain incomplete. This gap is particularly relevant given the complex and dynamic nature of biofilms, which involve coordinated processes such as initial adhesion, extracellular polymeric substances (EPS) matrix formation, and maturation.

In parallel, recent advances in biomaterials and bioengineering have opened new opportunities to translate lactobacilli-based antimicrobial strategies into practical applications [13]. However, the direct use of live bacteria is often limited by issues such as environmental instability, uncontrolled proliferation, and insufficient retention at target sites [14]. To address these challenges, emerging approaches have begun to integrate lactobacilli or their functional components into biomaterial platforms, such as hydrogels and encapsulation systems, enabling controlled delivery, enhanced stability, and sustained activity [15,16]. These biohybrid systems offer a promising avenue for the development of next-generation antimicrobial materials, particularly for the prevention and treatment of biofilm-associated infections.

In this review, we aim to provide a comprehensive and component-oriented overview of lactobacilli-derived antimicrobial and anti-biofilm strategies. We first categorize the functional components of lactobacilli, including secreted factors, cell-associated molecules, and whole-cell effects. We then systematically discuss the molecular mechanisms underlying their antimicrobial activities, with a particular focus on their roles in biofilm inhibition and disruption. Finally, we highlight emerging biomaterial-based approaches that leverage lactobacilli or their derivatives for translational applications. By bridging molecular functions with material-level innovations, this review seeks to provide new insights into the development of lactobacilli-based antimicrobial platforms for combating biofilm-related challenges.

## 2. Functional Components of Lactobacillus-Derived Antimicrobial Systems

**Figure 1:**
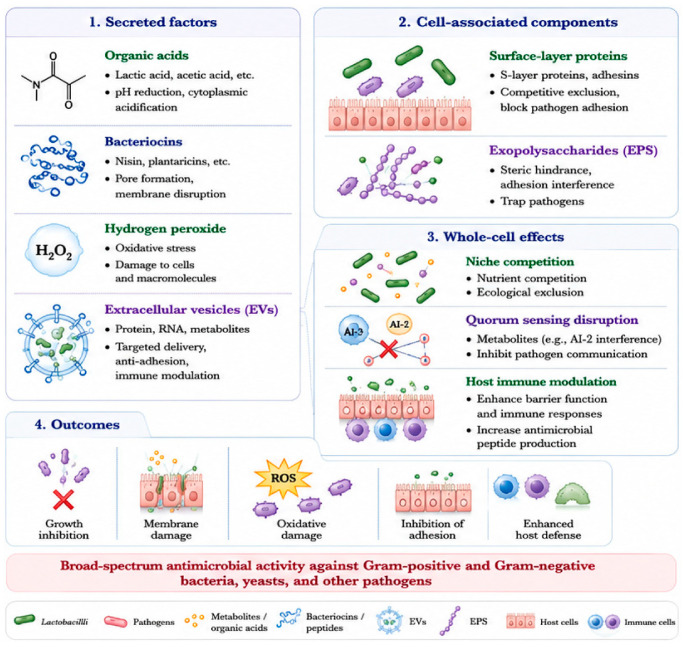
Antimicrobial mechanisms of LAB and their functional components. Schematic overview of the major antimicrobial mechanisms mediated by LAB-derived factors. Secreted antimicrobial components, including organic acids, bacteriocins, hydrogen peroxide, biosurfactants, extracellular vesicles (EVs), and exopolysaccharides (EPS), contribute to pathogen inhibition through multiple mechanisms such as cytoplasmic acidification, membrane disruption, oxidative stress induction, anti-adhesion activity, and targeted delivery of bioactive molecules. Cell-associated structures, including surface-layer (S-layer) proteins and adhesins, mediate competitive exclusion and interference with pathogen attachment. In addition, whole-cell effects, such as niche competition, quorum sensing disruption, and host immune modulation, further contribute to antimicrobial activity. Collectively, these mechanisms enable broad-spectrum inhibition of Gram-positive and Gram-negative bacteria, fungi, and other pathogenic microorganisms. This figure was generated with AI-assisted tools and subsequently reviewed and validated by the authors.

Lactobacilli-mediated antimicrobial activity is not governed by a single dominant factor but rather arises from a coordinated network of functional components operating across multiple biological levels. These include cell-free secreted antimicrobial molecules, cell-associated structures, and whole-cell ecological behaviors, which together enable both direct inhibition of pathogens and indirect modulation of the surrounding microenvironment. Increasing evidence suggests that these components function synergistically, allowing lactobacilli to adapt to diverse ecological niches and effectively suppress pathogenic microorganisms, particularly in the context of biofilm-associated infections (Figure 1; Table 1).

### 2.1. Cell-Free Secreted Antimicrobial Factors

Among the most extensively characterized antimicrobial mechanisms of lactobacilli are the production of extracellular metabolites and bioactive molecules. Organic acids, particularly lactic acid and acetic acid, represent the primary metabolic outputs and play a central role in pathogen inhibition [17,18,19]. Beyond lowering environmental pH, these acids can diffuse across bacterial membranes in their undissociated forms and dissociate intracellularly, leading to cytoplasmic acidification and metabolic disruption. For instance, lactic acid produced by *Lactiplantibacillus plantarum* has been shown to inhibit the growth of *E. coli* and *Salmonella enterica* through intracellular acid stress [20,21], while in the vaginal microbiota, acidification mediated by *Lactobacillus crispatus* contributes significantly to the suppression of pathogens such as *Gardnerella vaginalis* [22].

In addition to small-molecule metabolites, many lactobacilli species produce bacteriocins, a diverse class of ribosomally synthesized antimicrobial peptides [52]. These peptides typically exert their effects through membrane permeabilization or pore formation, although some also interfere with intracellular processes [53]. Well-characterized examples include nisin, which disrupts bacterial membranes and is widely used as a food preservative [54], and plantaricins produced by *L. plantarum*, which exhibit activity against pathogens such as *Listeria monocytogenes* and biofilm-forming *S. aureus* [23]. Notably, certain bacteriocins have also been reported to penetrate biofilm structures, enhancing their relevance in biofilm-associated infections [24,25].

Hydrogen peroxide production represents another important antimicrobial mechanism, particularly under aerobic or microaerophilic conditions. Several lactobacilli species, including *Lactobacillus jensenii*, generate H_2_O_2_, which induces oxidative stress in susceptible microorganisms [28]. This mechanism has been implicated in the inhibition of pathogens such as *Neisseria gonorrhoeae* and *Candida albicans*, highlighting its relevance in mucosal environments [28,29].

More recently, extracellular vesicles (EVs) have emerged as an additional layer of antimicrobial functionality. These nanoscale, membrane-bound structures can encapsulate proteins, lipids, and nucleic acids, enabling the protected and targeted delivery of bioactive molecules. For example, EVs derived from *Lacticaseibacillus rhamnosus GG* have been shown to modulate host responses and interfere with pathogen adhesion, suggesting that EV-mediated communication extends the functional reach of lactobacilli beyond direct cell-to-cell interactions [30,31].

In parallel, certain lactobacilli strains produce biosurfactants that reduce surface tension and alter physicochemical properties of microbial interfaces. These molecules have been demonstrated to inhibit the adhesion of pathogens such as *S. aureus* and *P. aeruginosa*, and in some cases, to disrupt pre-formed biofilms by modifying surface hydrophobicity [35,36]. Together, these secreted factors constitute a chemically diverse arsenal that underpins the direct antimicrobial capacity of lactobacilli.

### 2.2. Cell-Associated Components

In addition to cell-free secreted molecules, lactobacilli employ a range of cell surface-associated structures that contribute to antimicrobial activity primarily through physical interactions with pathogens and host surfaces. These components are particularly important during the early stages of microbial colonization and biofilm formation.

Surface-layer (S-layer) proteins, which form highly ordered crystalline arrays on the bacterial surface, play key roles in mediating adhesion and competitive interactions. For example, S-layer proteins from *Lactobacillus helveticus* have been shown to inhibit the attachment of *E. coli O157:H7* to intestinal epithelial cells, thereby reducing pathogen colonization [39,40,41]. Similarly, adhesion-associated proteins, including mucus-binding proteins found in species such as *Limosilactobacillus reuteri*, enable strong attachment to mucosal surfaces and facilitate competitive exclusion of pathogens such as *Salmonella* and *Clostridioides difficile* [42,43,55].

Exopolysaccharides represent another important class of cell-associated components with multifunctional roles. These polymers can modulate surface properties, promote bacterial aggregation, and interfere with pathogen adhesion. In some cases, exopolysaccharides derived from *L. plantarum* has been shown to inhibit biofilm formation by pathogens such as *E. coli* and *P. aeruginosa*, potentially through interference with extracellular matrix assembly [32,33]. Given their structural similarity to components of pathogenic biofilms, exopolysaccharides may also interact directly with biofilm matrices, an aspect that is increasingly recognized but remains incompletely understood.

### 2.3. Whole-Cell and Community-Level Effects

Beyond individual molecular components, the antimicrobial activity of lactobacilli is strongly shaped by whole-cell behaviors and ecological interactions within microbial communities. These effects reflect the ability of lactobacilli to function as an active ecological competitor rather than merely a passive producer of antimicrobial compounds.

Nutrient competition represents a fundamental mechanism, whereby lactobacilli limits the availability of essential resources required for pathogen growth [46]. For example, in the gastrointestinal tract, efficient consumption of simple carbohydrates by lactobacilli can restrict the proliferation of enteric pathogens such as *Salmonella* [47]. In parallel, niche occupation and spatial competition further enhance this effect, as stable colonization of mucosal surfaces by lactobacilli physically limits access of pathogens to adhesion sites [48]. This phenomenon is well illustrated in the vaginal microbiota, where dominance of lactobacilli species is associated with reduced colonization by pathogens such as *G. vaginalis* [48].

Interference with quorum sensing (QS) systems represents another important layer of antimicrobial activity. Certain metabolites produced by lactobacilli have been shown to disrupt QS signaling pathways, including AI-2-mediated communication in *E. coli*, thereby reducing coordinated behaviors such as virulence factor production and biofilm formation [49,50].

Finally, lactobacilli can exert indirect antimicrobial effects through modulation of host immune responses. For instance, *L. rhamnosus GG* has been shown to stimulate the production of antimicrobial peptides and enhance epithelial barrier function, contributing to host-mediated defense against invading pathogens [51]. These interactions highlight the multifaceted nature of lactobacilli antimicrobial strategies, which extend beyond direct bacterial inhibition to include modulation of the host environment.

## 3. Anti-Biofilm Mechanism of *Lactobacillus*

Biofilm formation is a dynamic and multi-stage process involving initial adhesion, extracellular matrix development, and maturation. The anti-biofilm activity of lactobacilli extends beyond simple growth inhibition and involves coordinated physicochemical, molecular, and ecological mechanisms that interfere with multiple stages of biofilm development, from initial attachment to the destabilization of mature biofilms [56,57]. This stage-specific perspective provides a conceptual framework for the rational design of lactobacilli-based anti-biofilm strategies and their integration into advanced therapeutic and biomaterial systems.

### 3.1. Inhibition of Initial Adhesion

The initial attachment of planktonic bacteria to biotic or abiotic surfaces represents a critical step in biofilm formation and a primary target for lactobacilli-mediated interference. At this stage, cell-associated components and secreted molecules act synergistically to reduce pathogen adhesion. One major mechanism involves surface competition and receptor blocking, whereby lactobacilli occupy binding sites that would otherwise be accessible to pathogens [58]. For example, mucus-binding proteins expressed by *L. reuteri* enable strong adherence to intestinal mucosa, effectively limiting the attachment of pathogens such as *Salmonella* [44]. Similarly, S-layer proteins from *L. helveticus* have been shown to inhibit the adhesion of *E. coli O157:H7* to epithelial cells, highlighting the role of surface-associated structures in competitive exclusion [45].

In parallel, lactobacilli-derived biosurfactants contribute to anti-adhesion activity by altering the physicochemical properties of surfaces. These molecules reduce surface tension and disrupt hydrophobic interactions that are essential for bacterial attachment. For instance, biosurfactants produced by *Lactobacillus acidophilus* have been reported to inhibit adhesion of *S. aureus* and *P. aeruginosa* to both epithelial and abiotic surfaces [37,59].

Environmental modification further reinforces these effects. Organic acid production lowers local pH, which can influence the expression of adhesion-related genes in pathogens [38]. In vaginal ecosystems, lactic acid produced by *L. crispatus* maintains an acidic environment that suppresses colonization by *G. vaginalis* [22]. Additionally, exopolysaccharides produced by *Lactobacillus* may provide steric hindrance, physically blocking receptor–ligand interactions required for pathogen attachment. exopolysaccharides from *L. plantarum*, for example, has been shown to reduce adhesion of *E. coli* to surfaces [26]. Collectively, these mechanisms converge to prevent the transition from planktonic growth to surface-associated colonization, thereby inhibiting the earliest stage of biofilm development.

### 3.2. Early Biofilm Development Inhibition

**Figure 2:**
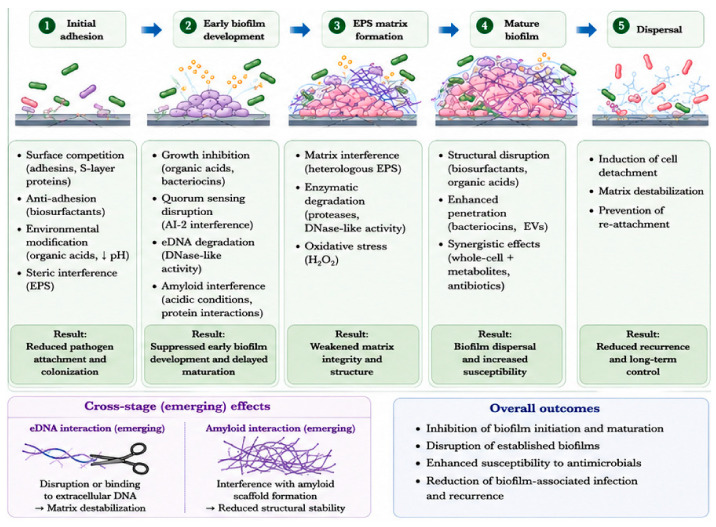
Stage-specific anti-biofilm mechanisms of *LAB*. Schematic illustration of how LAB interferes with distinct stages of biofilm development through multiple coordinated mechanisms. During initial adhesion, LAB-derived adhesins, S-layer proteins, biosurfactants, organic acids, and exopolysaccharides (EPS) reduce pathogen attachment through surface competition, anti-adhesion activity, environmental acidification, and steric interference. During early biofilm development, organic acids and bacteriocins inhibit bacterial proliferation, while quorum sensing interference disrupts biofilm-associated signaling pathways. Emerging mechanisms involving extracellular DNA (eDNA) degradation and modulation of amyloid aggregation may further impair matrix assembly and structural maturation. During EPS matrix formation and mature biofilm stages, LAB-derived factors destabilize biofilm architecture through matrix interference, enzymatic degradation, oxidative stress, enhanced antimicrobial penetration, and synergistic interactions with conventional antibiotics. This figure was generated with AI-assisted tools and subsequently reviewed and validated by the authors.

Following initial adhesion, bacterial cells proliferate and form microcolonies, a process that requires coordinated growth and intercellular communication. Lactobacilli interfere with this stage through both direct antimicrobial activity and disruption of QS systems. Secreted antimicrobial compounds, including organic acids and bacteriocins, play a central role in suppressing bacterial proliferation within early biofilms (Figure 2; Table 2). For example, plantaricins produced by *L. plantarum* exhibit inhibitory activity against pathogens such as *Listeria monocytogenes*, limiting the expansion of microcolonies [27]. In addition to direct growth inhibition, bacteriocins can compromise membrane integrity, reducing bacterial viability within developing biofilms [34].

Equally important is the ability of lactobacilli to interfere with QS signaling pathways that regulate biofilm-associated gene expression. Certain metabolites produced by lactobacilli have been shown to disrupt AI-2-mediated communication in bacteria such as *E. coli*, leading to reduced expression of virulence factors and impaired coordination of biofilm formation [50]. By targeting QS systems, lactobacilli effectively weaken the collective behavior required for stable colony formation.

These combined effects—growth inhibition and communication disruption—limit the structural and functional maturation of early biofilms, preventing their progression into more complex and resistant forms. Recent studies have highlighted the critical role of eDNA and amyloid-like proteins as structural components of biofilm matrices [56]. These elements contribute to matrix stability, intercellular adhesion, and resistance to environmental stress, making them attractive targets for anti-biofilm strategies. Mechanistically, eDNA can function as a nucleation center that promotes the assembly of amyloid proteins and extracellular polysaccharides, thereby initiating matrix formation [60]. In turn, amyloid fibrils form highly ordered, β-sheet-rich structures that enhance the mechanical strength and integrity of the biofilm matrix [61]. Notably, the eDNA–amyloid interaction framework discussed here was originally proposed in our previous work examining microbial amyloid biology. Its extension to lactobacilli-mediated biofilm disruption remains hypothetical, as direct experimental evidence in Lactobacillus species is currently limited. Nevertheless, this framework may provide a useful conceptual basis for future studies investigating the interaction between extracellular DNA, amyloid-like structures, and biofilm stability.

Although direct evidence remains limited, emerging data suggest that lactobacilli-derived factors may interact with eDNA and amyloid structures. For example, DNase-like activities have been reported in certain LAB strains [63], raising the possibility that LAB-derived nucleases may influence eDNA dynamics within biofilm matrices. However, it remains to be established whether these activities represent truly secreted extracellular DNases capable of degrading biofilm-associated eDNA, rather than intracellular enzymes released following bacterial cell lysis. In addition, the acidic microenvironment generated by lactobacilli metabolism may further modulate amyloid formation, as reduced pH conditions have been reported to delay or alter the aggregation kinetics of amyloid proteins [62]. Together, these effects suggest that lactobacilli may interfere with the assembly and stabilization of biofilm matrices, ultimately impairing biofilm maturation and limiting its formation.

Given the growing recognition of eDNA–protein interactions, including amyloid–DNA complexes, as key determinants of biofilm architecture, it is plausible that lactobacilli interfere with these interactions through both enzymatic and physicochemical mechanisms. While this area remains largely unexplored, it represents a promising direction for future research, particularly in the context of developing targeted anti-biofilm interventions.

### 3.3. Mature Biofilm Disruption

Mature biofilms exhibit high levels of structural complexity and resistance to antimicrobial agents, making them particularly difficult to eradicate. Notably, lactobacilli have demonstrated the capacity to disrupt established biofilms through multiple complementary mechanisms.

Biosurfactants and organic acids play important roles in structural disruption and biofilm dispersal. By altering surface properties and weakening intercellular interactions, these molecules can promote detachment of biofilm-associated cells. For example, biosurfactants produced by lactobacilli species have been shown to disrupt established biofilms of *P. aeruginosa*, leading to reduced biomass and increased susceptibility to external stress [64,65].

Another key mechanism involves enhanced penetration of antimicrobial agents. Bacteriocins and EVs can facilitate the delivery of bioactive molecules into the interior of biofilms, overcoming diffusion limitations imposed by the matrix [66]. EVs derived from *L. rhamnosus GG*, for instance, have been reported to transport functional cargo capable of modulating microbial and host responses, suggesting a role in targeting biofilm-embedded cells.

Importantly, lactobacilli can also act synergistically with conventional antibiotics [67,68]. Co-treatment with LAB or their metabolites has been shown to increase antibiotic sensitivity in biofilm-forming bacteria, indicating that lactobacilli-mediated matrix disruption and metabolic interference may enhance the efficacy of existing antimicrobial therapies.

## 4. From Biology to Materials: Lactobacilli-Based Biomaterial Applications

While the antimicrobial and anti-biofilm activities of lactobacilli have been extensively characterized at the molecular and cellular levels, translating these biological functions into practical applications remains a significant challenge. In particular, many of the functional components described above are subject to rapid diffusion, environmental instability, and limited retention at target sites, which can restrict their effectiveness in real-world settings. These limitations highlight the need for strategies that can preserve, localize, and control the activity of lactobacilli-derived antimicrobial systems. In this context, the integration of lactobacilli with biomaterial platforms has emerged as a promising approach to bridge the gap between biological function and translational application.

### 4.1. Rationale for Biomaterial Integration

Despite the well-documented antimicrobial and anti-biofilm activities of lactobacilli, the direct application of live bacteria or their metabolites remains limited by several practical challenges. These include poor environmental stability, rapid loss of viability under non-optimal conditions, uncontrolled proliferation, and insufficient retention at target sites. Such limitations are particularly problematic in settings where sustained and localized antimicrobial activity is required, such as chronic wound infections, implant-associated biofilms, or mucosal surfaces.

Moreover, many of the functional components produced by lactobacilli, including organic acids, bacteriocins, and biosurfactants, are subject to rapid diffusion and dilution in physiological environments. As a result, their effective concentrations may fall below effective biological concentrations required for antimicrobial activity, thereby reducing their functional efficacy. These constraints highlight a fundamental gap between the intrinsic biological potential of lactobacilli and its translational application.

To address these challenges, increasing attention has been directed toward the integration of lactobacilli or their functional components into biomaterial platforms. Such approaches aim to provide structural support, protect bacterial viability, and enable controlled release of antimicrobial factors, thereby bridging the gap between biological function and practical application.

### 4.2. Hydrogel-Based Delivery Systems

Among various biomaterial platforms, hydrogels have emerged as particularly promising systems for the delivery and functionalization of lactobacilli [69]. Hydrogels are three-dimensional, water-rich polymer networks that can encapsulate living cells while maintaining a biocompatible microenvironment [70]. Their high water content, tunable mechanical properties, and permeability make them well-suited for applications involving microbial systems.

Beyond alginate, other hydrogel systems, including chitosan-based, alginage-based, and hybrid hydrogels, have been explored to further tailor mechanical strength, degradation profiles, and functional properties [71]. These materials can be engineered to respond to environmental stimuli, such as pH or enzymatic activity, enabling responsive or on-demand release of antimicrobial agents. Hydrogel-based delivery systems have emerged as promising platforms for improving the stability and functionality of probiotic-derived antimicrobial factors. Among these materials, alginate remains the most extensively investigated food-grade hydrogel owing to its biocompatibility, mild gelation conditions, and ability to provide protective microenvironments for encapsulated bacteria.

Recent studies have demonstrated that alginate encapsulation can enhance probiotic viability, regulate bacterial release, and improve resistance to environmental stresses. For example, calcium alginate beads were shown to maintain higher long-term viability of encapsulated Lacticaseibacillus paracasei compared with conventional liquid cultures, while exhibiting controlled release behavior, with less than 1% bacterial release during the first 12 h and approximately 8% release after 24 h. Encapsulation also improved bacterial tolerance to oxidative stress and supported the growth of oxygen-sensitive Bifidobacterium species under aerobic conditions [72]. In addition to alginate alone, chitosan-containing hydrogel systems have attracted increasing interest. Chitosan coatings can enhance mechanical stability, reduce permeability, and improve retention of encapsulated probiotics. Recent studies have reported that alginate–chitosan composite hydrogels improve probiotic survival under gastrointestinal stress conditions while maintaining antimicrobial activity, highlighting their potential as next-generation probiotic delivery platforms [73].

Collectively, current evidence suggests that alginate-based and alginate–chitosan composite hydrogels represent the most well-established hydrogel systems for probiotic encapsulation, whereas additional studies are still required to evaluate other biomaterial platforms and their suitability for antimicrobial applications.

Importantly, the physicochemical properties of hydrogel materials strongly influence the viability, metabolic activity, and release behavior of encapsulated lactobacilli. Parameters such as porosity, mechanical strength, oxygen permeability, swelling behavior, and biodegradability can directly affect bacterial survival and functional performance. For example, highly porous hydrogels may facilitate nutrient diffusion and metabolite exchange, whereas dense polymer networks may improve retention and prolong localized antimicrobial activity [74]. In addition, different biomaterials exhibit distinct advantages in terms of biocompatibility, stability, responsiveness, and ease of fabrication, making material selection a critical consideration for designing lactobacilli-based delivery systems.

### 4.3. Application in Biofilm-Associated Contexts

The convergence of lactobacilli biology and biomaterial engineering has led to a range of emerging applications, particularly in biofilm-associated environments where conventional treatments are often insufficient.

In wound management, hydrogel systems containing lactobacilli or their metabolites have shown potential in preventing infection and promoting healing by inhibiting pathogenic biofilms and modulating local immune responses [75]. In gastrointestinal applications, encapsulated probiotics can enhance survival through the upper digestive tract and enable targeted delivery to the intestine, where they can modulate microbiota composition and suppress pathogen colonization [76,77].

Similarly, in the oral cavity, biomaterial-based delivery systems may be used to control biofilm formation associated with dental caries and periodontal disease [78,79,80]. The ability of lactobacilli to interfere with adhesion and disrupt established biofilms makes it particularly suitable for incorporation into coatings, gels, or slow-release devices targeting oral pathogens such as *Streptococcus mutans*.

In addition, anti-biofilm coatings for medical devices and implants represent another promising application area [81]. Incorporating lactobacilli-derived components into surface coatings may reduce microbial colonization and biofilm formation, addressing a major cause of device-associated infections.

### 4.4. Toward Living Antimicrobial Materials

The integration of lactobacilli into biomaterials represents a shift from passive antimicrobial systems toward living antimicrobial materials, in which biological activity is maintained and dynamically regulated. In such systems, encapsulated bacteria function as continuous producers of antimicrobial compounds, rather than as static agents.

This concept offers several advantages over conventional antimicrobial approaches. First, sustained in situ production of antimicrobial factors can maintain effective local concentrations without the need for repeated administration. Second, the ability of living cells to respond to environmental cues allows for adaptive regulation of antimicrobial activity. For instance, bacterial metabolism may be upregulated in response to the presence of competing microorganisms, leading to increased production of inhibitory compounds.

Furthermore, the combination of multiple functional mechanisms—such as organic acid production, bacteriocin secretion, and biofilm disruption—within a single living system enables multimodal antimicrobial activity, which is particularly advantageous for targeting complex biofilm structures. When integrated into biomaterials, these effects can be spatially confined and temporally controlled, enhancing both efficacy and safety.

Recent advances have also explored the incorporation of engineered or selected lactobacilli strains with enhanced functional properties, as well as the co-delivery of bacteria with complementary biomolecules [82,83,84]. Such approaches further expand the design space of living antimicrobial materials and open new possibilities for precision antimicrobial interventions.

## 5. Challenges and Future Perspectives

Despite the growing body of evidence supporting the antimicrobial and anti-biofilm potential of lactobacilli, several critical challenges remain that limit its full translational and mechanistic exploitation. Addressing these limitations will be essential for advancing lactobacilli-based strategies from experimental systems toward clinically and industrially relevant applications.

One of the primary challenges lies in the complexity and heterogeneity of lactobacilli-mediated antimicrobial activity. While numerous studies have identified individual factors such as organic acids, bacteriocins, and exopolysaccharides, their relative contributions and potential synergistic interactions remain poorly defined. Most current studies focus on single strains or isolated components, making it difficult to establish a unified framework that links specific molecular factors to functional outcomes. In addition, strain-specific variability represents a significant barrier to generalization. Even closely related lactobacilli strains can exhibit markedly different antimicrobial profiles, reflecting differences in metabolic capacity, surface structures, and regulatory networks [85]. This heterogeneity complicates both mechanistic interpretation and practical application, highlighting the need for systematic, comparative approaches.

Future research should therefore move toward multi-dimensional characterization strategies, integrating genomics, transcriptomics, metabolomics, and phenotypic assays to map functional outputs to specific molecular determinants. Such approaches will enable the identification of key effectors and facilitate the rational selection or engineering of strains with optimized antimicrobial properties. A key direction in the field is the transition from empirical use of lactobacilli toward mechanistically guided and programmable antimicrobial systems. This shift will require deeper integration of computational and experimental approaches. Artificial intelligence (AI) and machine learning-based models have the potential to accelerate the identification of functional strains and antimicrobial molecules by analyzing large-scale datasets derived from microbial genomes and metabolomes. Such approaches could enable predictive screening of strains with specific anti-biofilm properties or the design of optimized microbial consortia.

Another major challenge concerns the lack of standardization across experimental systems. Variations in culture conditions, strain selection, assay design, and biofilm models often lead to inconsistent or non-comparable results. For example, differences in medium composition or oxygen availability can significantly influence the production of antimicrobial metabolites, thereby affecting experimental outcomes [84,85]. Similarly, biofilm studies frequently rely on simplified in vitro models that may not accurately reflect the complexity of environments in vivo. This discrepancy limits the translational relevance of many findings and underscores the need for more physiologically relevant models, including multi-species biofilms and host-associated systems. To improve reproducibility and comparability, the development of standardized protocols and benchmarking frameworks will be critical. Establishing reference strains, unified assay conditions, and quantitative metrics for antimicrobial and anti-biofilm activity would greatly enhance the robustness of future studies.

From a translational perspective, several practical limitations hinder the application of lactobacilli-based antimicrobial systems. These include challenges related to bacterial viability, stability during storage and delivery, and controlled activity at target sites. Although biomaterial-based approaches, such as hydrogel encapsulation, have shown promise in addressing some of these issues, further optimization is required to balance bacterial survival, release kinetics, and functional output. Regulatory considerations also represent an important barrier, particularly for applications involving live microorganisms. Issues related to safety, strain characterization, and long-term effects must be carefully evaluated before clinical or industrial deployment. In addition, large-scale production and formulation of lactobacilli-based systems require cost-effective and scalable manufacturing processes. Advances in biomaterial engineering and synthetic biology offer potential solutions to these challenges. For example, the design of responsive or stimuli-triggered delivery systems may enable more precise spatial and temporal control of antimicrobial activity. Similarly, genetic engineering approaches could be used to enhance specific functional traits, such as bacteriocin production or biofilm-targeting capabilities.

Another promising direction involves the development of microbiome-informed strategies, in which lactobacilli are deployed as part of a broader ecological intervention. Rather than acting in isolation, lactobacilli could be used to reshape microbial communities and suppress pathogen colonization through community-level dynamics.

Importantly, emerging insights into biofilm matrix biology provide new opportunities for targeted intervention. In particular, eDNA and amyloid-like proteins have been increasingly recognized as key structural elements of biofilms. Understanding how lactobacilli-derived factors interact with these components may enable the development of matrix-targeted anti-biofilm strategies, such as disrupting eDNA–protein networks or interfering with amyloid scaffold formation. These approaches could complement existing antimicrobial mechanisms and provide new avenues for overcoming biofilm-associated resistance.

Although lactobacilli are generally regarded as safe and have a long history of use in food and probiotic applications, potential safety concerns should not be overlooked. Rare cases of Lactobacillus-associated bacteremia, endocarditis, liver abscesses, and other opportunistic infections have been reported, particularly in immunocompromised individuals, critically ill patients, premature infants, and patients with severe underlying diseases. Therefore, careful strain selection, genomic safety assessment, evaluation of antibiotic resistance profiles, and comprehensive risk–benefit analyses are essential before clinical implementation. Future studies should also establish standardized safety evaluation frameworks to facilitate the responsible translation of lactobacilli-based antimicrobial therapies into clinical practice.

## 6. Conclusions

Lactobacilli have emerged as a versatile and multifaceted platform for antimicrobial and anti-biofilm intervention. Unlike conventional antimicrobial strategies that rely on single-target mechanisms, lactobacilli exert their effects through an integrated network of functional components, including secreted metabolites, cell-associated structures, and community-level interactions. This multi-layered system enables not only direct inhibition of pathogenic microorganisms but also targeted interference with key stages of biofilm development, from initial adhesion to matrix destabilization and biofilm dispersal.

In this review, we have provided a component-oriented framework that links specific molecular factors to their underlying antimicrobial and anti-biofilm mechanisms. By organizing current knowledge across distinct functional categories and biofilm stages, we highlight how diverse lactobacilli-derived factors collectively contribute to biofilm control. In particular, emerging insights into matrix-associated elements such as eDNA and amyloid-like proteins suggest new mechanistic avenues for targeting biofilm stability, expanding the scope of lactobacilli-based interventions beyond conventional paradigms.

Importantly, the integration of lactobacilli with biomaterial systems represents a critical step toward translating its biological potential into practical applications. Hydrogel-based encapsulation and related approaches enable controlled delivery, enhanced stability, and sustained antimicrobial activity, paving the way for the development of living, adaptive antimicrobial systems. These advances support a conceptual shift from static antimicrobial agents to dynamic, biologically active platforms capable of continuous and responsive function.

Despite these advances, significant challenges remain, including mechanistic complexity, strain variability, and translational barriers. Addressing these issues will require interdisciplinary efforts that combine microbiology, materials science, and systems-level analysis. Future research aimed at mechanistically guided design, multi-omics integration, and programmable microbial systems is expected to further unlock the potential of lactobacilli.

Overall, lactobacilli-based antimicrobial strategies represent a promising and rapidly evolving field. By bridging molecular mechanisms with material-based innovation, these approaches offer new opportunities for combating biofilm-associated infections and advancing next-generation antimicrobial technologies.

## Figures and Tables

**Table 1:** Component-centered summary of major antimicrobial and anti-biofilm factors produced by lactobacilli and their mechanisms of action.

Component (Molecule/Factor)	Representative Source	Mechanism of Action	Target Pathogen	Biofilm-Related Effect	References
Lactic acid	*L. plantarum*, *L. crispatus*	Cytoplasmic acidification, environmental pH reduction	*E. coli*, *Salmonella enterica*, *Gardnerella vaginalis*	Inhibits growth, adhesion, and colonization	[17,18,19,20,21,22]
Plantaricins	*L. plantarum*	Membrane permeabilization and growth inhibition	*Listeria monocytogenes*, *S. aureus*	Suppresses microcolony formation	[23,24,25,26,27]
H_2_O_2_	*L. jensenii*	Oxidative stress induction	*Neisseria gonorrhoeae*, *Candida albicans*	Destabilizes microbial survival within biofilms	[28,29]
Protein/RNA cargo	*L. rhamnosus GG*	Targeted delivery of bioactive molecules	Multiple pathogens	Anti-adhesion and immune modulation	[30,31]
Polysaccharides	*L. plantarum*	Adhesion interference and matrix interaction	*E. coli*, *P. aeruginosa*	Biofilm inhibition and matrix destabilization	[32,33,34]
Lipopeptides/glycolipids	*L. acidophilus*	Surface tension reduction and hydrophobicity alteration	*S. aureus*, *P. aeruginosa*	Anti-adhesion and biofilm dispersal	[35,36,37,38]
Surface-associated proteins	*L. helveticus*	Competitive exclusion and receptor blocking	*E. coli* O157:H7	Prevents initial adhesion	[39,40,41]
Mucus-binding proteins	*L. reuteri*	Niche occupation and competitive adhesion	*Salmonella*, *C. difficile*	Reduces colonization	[42,43,44,45]
Nutrient competition	Multiple *Lactobacillus* spp.	Resource depletion and ecological competition	Enteric pathogens	Limits pathogen proliferation	[46,47,48,49]
AI-2 modulation	LAB metabolites	Disruption of bacterial communication	*E. coli* and mixed biofilms	Reduces virulence and biofilm maturation	[50,51]

**Table 2:** Process-centered overview of lactobacilli-mediated interference across different stages of biofilm development.

Biofilm Stage	Mechanism	Molecular Action	Representative Example	Functional Outcome	References
Initial adhesion	Surface competition	Occupation of binding sites on host or abiotic surfaces	*L. reuteri* mucus-binding proteins inhibit *Salmonella* adhesion	Reduced pathogen attachment and colonization	[39,40,41,42,43,44,45,55,59]
	Anti-adhesion activity	Reduction of surface tension and hydrophobic interactions	*L. acidophilus* biosurfactants inhibit *S. aureus* and *P. aeruginosa* adhesion	Prevention of early biofilm formation	[35,36,37,38]
	Environmental modification	Local pH reduction affecting adhesion-related gene expression	*L. crispatus* suppresses *G. vaginalis* colonization via acidification	Inhibition of pathogen colonization	[17,18,19,20,21,22,26]
	Steric interference	Physical blocking of receptor–ligand interactions	*L. plantarum* EPS reduces *E. coli* adhesion	Reduced surface attachment	[32,33]
Early biofilm development	Growth inhibition	Metabolic disruption and membrane damage	Plantaricins inhibit *Listeria monocytogenes* growth	Suppressed microcolony expansion	[23,24,25,34,52,53,54,60]
	Quorum sensing disruption	Interference with signaling pathways regulating virulence and biofilm genes	*Lactobacillus* spp. inhibit QS signaling in *E. coli*	Impaired coordination of biofilm formation	[50,51]
	eDNA-associated interference (emerging)	Degradation of extracellular DNA involved in matrix nucleation	DNase activity reported in *L. delbrueckii*	Impaired matrix assembly and stabilization	[57,61,62]
	Amyloid-associated interference (emerging)	Modulation of amyloid aggregation kinetics and scaffold formation	Low pH delays amyloid fibril assembly	Delayed biofilm maturation and reduced structural cohesion	[57,63,64]
EPS matrix formation	Matrix interference	Competitive interaction with pathogen EPS components	*L. plantarum* EPS disrupts *P. aeruginosa* matrix assembly	Weakened biofilm structure	[32,33,61]
	Enzymatic degradation	Breakdown of proteinaceous and eDNA matrix components	LAB supernatants reduce *S. aureus* biofilm biomass	Reduced matrix integrity	[57,62]
	Oxidative stress	Damage to matrix-associated cells and macromolecules	*L. jensenii* inhibits pathogens through ROS generation	Destabilization of biofilm environment	[28,29]
Mature biofilm	Structural disruption	Alteration of biofilm architecture and weakening of intercellular interactions	Biosurfactants disrupt pre-formed *P. aeruginosa* biofilms	Biofilm dispersal	[35,36,65,66]
	Enhanced penetration	Delivery of antimicrobial molecules into biofilm interior	EVs from *L. rhamnosus* GG transport bioactive cargo	Increased antimicrobial susceptibility	[30,67]
	Synergistic antimicrobial effects	Enhancement of antibiotic sensitivity and metabolic stress	LAB co-treatment enhances antibiotic efficacy	Improved biofilm eradication	[67,68]
Cross-stage effects	Ecological competition	Restriction of pathogen access to nutrients and adhesion sites	Stable *Lactobacilli* colonization suppresses pathogen persistence	Broad inhibition of biofilm establishment	[46,47,48,49]
	Host immune modulation	Induction of antimicrobial peptides and barrier enhancement	*L. rhamnosus* GG enhances epithelial defense	Indirect suppression of biofilm-associated pathogens	[51]

## Data Availability

No new data were created or analyzed in this study. Data sharing is not applicable to this article.

## References

[1] Sharma A., Katoch P., Shrivastava R. (2026). Bacterial biofilm conundrum: Insight into the frontiers of antibiotic resistance and state-of-the-art anti-biofilm interventions. Front. Cell. Infect. Microbiol..

[2] Mendhe S., Badge A., Ugemuge S., Chandi D. (2023). Impact of Biofilms on Chronic Infections and Medical Challenges. Cureus.

[3] Liu H.Y., Prentice E.L., Webber M.A. (2024). Mechanisms of antimicrobial resistance in biofilms. npj Antimicrob. Resist..

[4] Bernardi S., Anderson A., Macchiarelli G., Hellwig E., Cieplik F., Vach K., Al-Ahmad A. (2021). Subinhibitory Antibiotic Concentrations Enhance Biofilm Formation of Clinical *Enterococcus faecalis* Isolates. Antibiotics.

[5] Krzyżek P., Migdał P., Tusiewicz K., Zawadzki M., Szpot P. (2024). Subinhibitory concentrations of antibiotics affect development and parameters of *Helicobacter pylori* biofilm. Front. Pharmacol..

[6] Ng M., Epstein S.B., Callahan M.T., Piotrowski B.O., Simon G.L., Roberts A.D., Keiser J.F., Kaplan J.B. (2013). Induction of MRSA Biofilm by Low-Dose β-Lactam Antibiotics: Specificity, Prevalence and Dose-Response Effects. Dose Response.

[7] Kaplan J.B., Izano E.A., Gopal P., Karwacki M.T., Kim S., Bose J.L., Bayles K.W., Horswill A.R. (2012). Low levels of β-lactam antibiotics induce extracellular DNA release and biofilm formation in Staphylococcus aureus. mBio.

[8] Sailer F.C., Meberg B.M., Young K.D. (2003). beta-Lactam induction of colanic acid gene expression in Escherichia coli. FEMS Microbiol. Lett..

[9] Jeong D., Kim D.H., Song K.Y., Seo K.H. (2018). Antimicrobial and anti-biofilm activities of *Lactobacillus kefiranofaciens* DD2 against oral pathogens. J. Oral Microbiol..

[10] Zheng J., Wittouck S., Salvetti E., Franz C.M.A.P., Harris H.M.B., Mattarelli P., O’Toole P.W., Pot B., Vandamme P., Walter J. (2020). A taxonomic note on the genus Lactobacillus: Description of 23 novel genera, emended description of the genus Lactobacillus Beijerinck 1901, and union of Lactobacillaceae and Leuconostocaceae. Int. J. Syst. Evol. Microbiol..

[11] Duar R.M., Lin X.B., Zheng J., Martino M.E., Grenier T., Pérez-Muñoz M.E., Leulier F., Gänzle M., Walter J. (2017). Lifestyles in transition: Evolution and natural history of the genus Lactobacillus. FEMS Microbiol. Rev..

[12] Chaichana N., Singkhamanan K., Wonglapsuwan M., Chusri S., Pomwised R., Surachat K. (2026). From Fermentation to Function: Genomic Diversity and Probiotic Potential in the Reclassified *Lactobacillus* Lineage. Comput. Struct. Biotechnol. J..

[13] Sabio L., Day G.J., Salmeron-Sanchez M. (2026). Probiotic-Based Materials as Living Therapeutics. Adv. Mater..

[14] Zhu Y., Wang Z., Bai L., Deng J., Zhou Q. (2021). Biomaterial-based encapsulated probiotics for biomedical applications: Current status and future perspectives. Mater. Des..

[15] Sahoo D., Rodriguez E., Nguyen K., Chintapula U. (2025). Probiotic Bacteria as Therapeutics and Biohybrid Drug Carriers: Advances, Design Strategies, and Future Outlook. ACS Appl. Bio Mater..

[16] Niu M., Zhao L., Gong S., Liu X., Zheng C., Jiao J., Wang F., Wang L. (2025). Oral administration of probiotic spores-based biohybrid system for efficient attenuation of Salmonella Typhimurium-induced colitis. J. Nanobiotechnol..

[17] Ibrahim S.A., Ayivi R.D., Zimmerman T., Siddiqui S.A., Altemimi A.B., Fidan H., Esatbeyoglu T., Bakhshayesh R.V. (2021). Lactic Acid Bacteria as Antimicrobial Agents: Food Safety and Microbial Food Spoilage Prevention. Foods.

[18] Szczerbiec D., Piechocka J., Głowacki R., Torzewska A. (2022). Organic Acids Secreted by *Lactobacillus* spp. Isolated from Urine and Their Antimicrobial Activity against Uropathogenic Proteus mirabilis. Molecules.

[19] El-Garhi H.M., Abd-Elghany A.A., Ibrahim A.M.A., El-Aidie S.A.M., Castro-Muñoz R. (2026). Effect of Organic Acids on Pathogenic and Lactic Acid Bacteria in Directly Raw Milk Cheddar Cheese. Probiotics Antimicrob. Proteins.

[20] Potočnjak M., Pušić P., Frece J., Abram M., Janković T., Gobin I. (2017). Three New *Lactobacillus plantarum* Strains in the Probiotic Toolbox against Gut Pathogen *Salmonella enterica* Serotype Typhimurium. Food Technol. Biotechnol..

[21] Byakika S., Mukisa I.M., Mugabi R., Muyanja C. (2019). Antimicrobial Activity of Lactic Acid Bacteria Starters against Acid Tolerant, Antibiotic Resistant, and Potentially Virulent *E. coli* Isolated from a Fermented Sorghum-Millet Beverage. Int. J. Microbiol..

[22] Breshears L.M., Edwards V.L., Ravel J., Peterson M.L. (2015). Lactobacillus crispatus inhibits growth of Gardnerella vaginalis and Neisseria gonorrhoeae on a porcine vaginal mucosa model. BMC Microbiol..

[23] Todorov S.D. (2009). Bacteriocins from Lactobacillus plantarum—Production, genetic organization and mode of action: Produção, organização genética e modo de ação. Braz. J. Microbiol..

[24] Yang Y., Kong X., Niu B., Yang J., Chen Q. (2024). Differences in Biofilm Formation of *Listeria monocytogenes* and Their Effects on Virulence and Drug Resistance of Different Strains. Foods.

[25] Pei J., Huang Y., Ren T., Guo Y., Dang J., Tao Y., Zhang Y., Abd El-Aty A.M. (2022). The Antibacterial Activity Mode of Action of Plantaricin YKX against *Staphylococcus aureus*. Molecules.

[26] Oh S.E., Heo S., Kim M., Moon Y., Lee S., Park C., Sung H., Lee G., Kim J., Sung M.H. (2025). Synthetic Plantaricins Derived from *Lactiplantibacillus plantarum* KM2 Induce Cell Lysis of *Listeria monocytogenes*. J. Microbiol. Biotechnol..

[27] Kranjec C., Ovchinnikov K.V., Grønseth T., Ebineshan K., Srikantam A., Diep D.B. (2020). A bacteriocin-based antimicrobial formulation to effectively disrupt the cell viability of methicillin-resistant Staphylococcus aureus (MRSA) biofilms. npj Biofilms Microbiomes.

[28] Martín R., Suárez J.E. (2010). Biosynthesis and degradation of H_2_O_2_ by vaginal lactobacilli. Appl. Environ. Microbiol..

[29] Cobrado L., Ricardo E., Ramalho P., Fernandes A.R., Rodrigues A.G. (2023). Does repeated exposure to hydrogen peroxide induce Candida auris resistance?. Antimicrob. Resist. Infect. Control.

[30] Petrova M.I., Imholz N.C., Verhoeven T.L., Balzarini J., Van Damme E.J., Schols D., Vanderleyden J., Lebeer S. (2016). Lectin-like Molecules of Lactobacillus rhamnosus GG Inhibit Pathogenic Escherichia coli and Salmonella Biofilm Formation. PLoS ONE.

[31] Domínguez Rubio A.P., D’Antoni C.L., Piuri M., Pérez O.E. (2022). Probiotics, Their Extracellular Vesicles and Infectious Diseases. Front. Microbiol..

[32] Pradeepa, Shetty A.D., Matthews K., Hegde A.R., Akshatha B., Mathias A.B., Mutalik S., Vidya S.M. (2016). Multidrug resistant pathogenic bacterial biofilm inhibition by Lactobacillus plantarum exopolysaccharide. Bioact. Carbohydr. Diet. Fibre.

[33] Reid G., Burton J. (2002). Use of Lactobacillus to prevent infection by pathogenic bacteria. Microbes Infect..

[34] Wang S., Liu X., Liu H., Zhang L., Guo Y., Yu S., Wozniak D.J., Ma L.Z. (2015). The exopolysaccharide Psl-eDNA interaction enables the formation of a biofilm skeleton in Pseudomonas aeruginosa. Environ. Microbiol. Rep..

[35] De Giani A., Zampolli J., Di Gennaro P. (2021). Recent Trends on Biosurfactants with Antimicrobial Activity Produced by Bacteria Associated with Human Health: Different Perspectives on Their Properties, Challenges, and Potential Applications. Front. Microbiol..

[36] Kim M., Khatun J., Khan F., Kim Y.M. (2026). Lactic Acid Bacteria as Natural Antimicrobials: Biofilm Control in Food and Food Industry. Antibiotics.

[37] Adnan M., Siddiqui A.J., Noumi E., Ashraf S.A., Awadelkareem A.M., Hadi S., Snoussi M., Badraoui R., Bardakci F., Sachidanandan M. (2023). Biosurfactant derived from probiotic Lactobacillus acidophilus exhibits broad-spectrum antibiofilm activity and inhibits the quorum sensing-regulated virulence. Biomol. Biomed..

[38] Yang S., Xu X., Peng Q., Ma L., Qiao Y., Shi B. (2023). Exopolysaccharides from lactic acid bacteria, as an alternative to antibiotics, on regulation of intestinal health and the immune system. Anim. Nutr..

[39] de la Fuente-Núñez C., Mertens J., Smit J., Hancock R.E. (2012). The bacterial surface layer provides protection against antimicrobial peptides. Appl. Environ. Microbiol..

[40] Acosta M.P., Ruzal S.M., Cordo S.M. (2016). S-layer proteins from Lactobacillus sp. inhibit bacterial infection by blockage of DC-SIGN cell receptor. Int. J. Biol. Macromol..

[41] Baillo A.A., Albarracín L., Heredia Ojeda E., Elean M., Gong W., Kitazawa H., Villena J., Fadda S. (2026). Secretome Profiling of *Lactiplantibacillus plantarum* CRL681 Predicts Potential Molecular Mechanisms Involved in the Antimicrobial Activity Against *Escherichia coli* O157:H7. Antibiotics.

[42] Shi S., Qi Z., Sheng T., Tu J., Shao Y., Qi K. (2019). Antagonistic trait of Lactobacillus reuteri S5 against Salmonella enteritidis and assessment of its potential probiotic characteristics. Microb. Pathog..

[43] Engevik M.A., Danhof H.A., Shrestha R., Chang-Graham A.L., Hyser J.M., Haag A.M., Mohammad M.A., Britton R.A., Versalovic J., Sorg J.A. (2020). Reuterin disrupts *Clostridioides difficile* metabolism and pathogenicity through reactive oxygen species generation. Gut Microbes.

[44] Jensen H., Roos S., Jonsson H., Rud I., Grimmer S., van Pijkeren J.P., Britton R.A., Axelsson L. (2014). Role of Lactobacillus reuteri cell and mucus-binding protein A (CmbA) in adhesion to intestinal epithelial cells and mucus in vitro. Microbiology.

[45] Taverniti V., Stuknyte M., Minuzzo M., Arioli S., De Noni I., Scabiosi C., Cordova Z.M., Junttila I., Hämäläinen S., Turpeinen H. (2013). S-layer protein mediates the stimulatory effect of Lactobacillus helveticus MIMLh5 on innate immunity. Appl. Environ. Microbiol..

[46] Froebel L.K., Froebel L.E., Duong T. (2020). Refined functional carbohydrates reduce adhesion of Salmonella and Campylobacter to poultry epithelial cells in vitro. Poult. Sci..

[47] Yue Y., Xu X., Yang B., Lu J., Zhang S., Liu L., Nassar K., Zhang C., Zhang M., Pang X. (2020). Stable Colonization of Orally Administered *Lactobacillus casei* SY13 Alters the Gut Microbiota. BioMed Res. Int..

[48] Xu H., Zhang S., Zhang B., Jiang N., Xu Y., Chen X., Han L. (2025). Vaginal colonization of Lactobacilli: Mechanism and function. Microb. Pathog..

[49] Liu X., Liu Q., Sun S., Sun H., Wang Y., Shen X., Zhang L. (2022). Exploring AI-2-mediated interspecies communications within rumen microbial communities. Microbiome.

[50] Meng F., Zhao M., Lu Z. (2022). The LuxS/AI-2 system regulates the probiotic activities of lactic acid bacteria. Trends Food Sci. Technol..

[51] Yan F., Polk D.B. (2012). *Lactobacillus rhamnosus* GG: An Updated Strategy to Use Microbial Products to Promote Health. Funct. Food Rev..

[52] Hassan M.U., Nayab H., Rehman T.U., Williamson M.P., Haq K.U., Shafi N., Shafique F. (2020). Characterisation of Bacteriocins Produced by *Lactobacillus* spp. Isolated from the Traditional Pakistani Yoghurt and Their Antimicrobial Activity against Common Foodborne Pathogens. BioMed Res. Int..

[53] Kumariya R., Garsa A.K., Rajput Y.S., Sood S.K., Akhtar N., Patel S. (2019). Bacteriocins: Classification, synthesis, mechanism of action and resistance development in food spoilage causing bacteria. Microb. Pathog..

[54] Shin J.M., Gwak J.W., Kamarajan P., Fenno J.C., Rickard A.H., Kapila Y.L. (2016). Biomedical applications of nisin. J. Appl. Microbiol..

[55] Mahdhi A., Leban N., Chakroun I., Bayar S., Mahdouani K., Majdoub H., Kouidhi B. (2018). Use of extracellular polysaccharides, secreted by Lactobacillus plantarum and Bacillus spp., as reducing indole production agents to control biofilm formation and efflux pumps inhibitor in Escherichia coli. Microb. Pathog..

[56] Gong W., Cheng X., Villena J., Kitazawa H. (2025). eDNA-Amyloid Synergistic Interactions in Bacterial Biofilms: A Hidden Driver of Antimicrobial Resistance. Int. J. Mol. Sci..

[57] Kang S., Yang Y., Hou W., Zheng Y. (2024). Inhibitory Effects of Lactobionic Acid on Biofilm Formation and Virulence of *Staphylococcus aureus*. Foods.

[58] Muscariello L., De Siena B., Marasco R. (2020). Lactobacillus Cell Surface Proteins Involved in Interaction with Mucus and Extracellular Matrix Components. Curr. Microbiol..

[59] Walencka E., Rózalska S., Sadowska B., Rózalska B. (2008). The influence of Lactobacillus acidophilus-derived surfactants on staphylococcal adhesion and biofilm formation. Folia Microbiol..

[60] Hobley L., Harkins C., MacPhee C.E., Stanley-Wall N.R. (2015). Giving structure to the biofilm matrix: An overview of individual strategies and emerging common themes. FEMS Microbiol. Rev..

[61] Peril M.A., Auad L., Raya R.R. (2000). Deoxyribonuclease activities in Lactobacillus delbrueckii. Microbiol. Res..

[62] Abán C.L., Orosco S., Argañaraz Aybar J.N., Albarracín L., Venecia A., Perret L., Ortiz Mayor S., Nishiyama K., Valdéz J.C., Kitazawa H. (2024). Effect of *Lactiplantibacillus plantarum* cell-free culture on bacterial pathogens isolated from cystic fibrosis patients: In vitro and in vivo studies. Front. Microbiol..

[63] Pfefferkorn C.M., McGlinchey R.P., Lee J.C. (2010). Effects of pH on aggregation kinetics of the repeat domain of a functional amyloid, Pmel17. Proc. Natl. Acad. Sci. USA.

[64] Renye J.A., Chen C.Y., Miller A., Lee J., Oest A., Lynn K.J., Felton S.M., Guragain M., Tomasula P.M., Berger B.W. (2025). Integrating Bacteriocins and Biofilm-Degrading Enzymes to Eliminate *L. monocytogenes* Persistence. Int. J. Mol. Sci..

[65] Liévin-Le Moal V., Servin A.L. (2014). Anti-infective activities of lactobacillus strains in the human intestinal microbiota: From probiotics to gastrointestinal anti-infectious biotherapeutic agents. Clin. Microbiol. Rev..

[66] Lee J.H., Kim J., Kim G.Y. (2023). Synergistic Effects of a Probiotic Culture Extract and Antimicrobial Combinations against Multidrug-Resistant *Acinetobacter baumannii*. Medicina.

[67] Yu S., Sun H., Li Y., Wei S., Xu J., Liu J. (2022). Hydrogels as promising platforms for engineered living bacteria-mediated therapeutic systems. Mater. Today Bio.

[68] Ho T.C., Chang C.C., Chan H.P., Chung T.W., Shu C.W., Chuang K.P., Duh T.H., Yang M.H., Tyan Y.C. (2022). Hydrogels: Properties and Applications in Biomedicine. Molecules.

[69] Parvin N., Kumar V., Joo S.W., Mandal T.K. (2024). Cutting-Edge Hydrogel Technologies in Tissue Engineering and Biosensing: An Updated Review. Materials.

[70] Annabi N., Nichol J.W., Zhong X., Ji C., Koshy S., Khademhosseini A., Dehghani F. (2010). Controlling the porosity and microarchitecture of hydrogels for tissue engineering. Tissue Eng. Part. B Rev..

[71] Li X., Fan Y., Li J., Yan C., Wang P., Jia C. (2026). Antibacterial hydrogels for skin infected wounds: Frontier approaches as antibiotic alternatives therapy. Front. Cell. Infect. Microbiol..

[72] Gong W., Arellano-Arriagada L., Villena J., Nishiyama K., Kitazawa H. (2026). Encapsulation in Calcium Alginate Beads Regulates Growth, Release and Viability of Probiotic Bacteria Through Protective Microenvironments. Gels.

[73] Liu Y., Guo M., Lu J., Zhao J., Wang G., Zhang R., Zhang H., Zhang Z. (2026). Biocompatible Chitosan-Based Nanoparticles for the Delivery of Lactobacillus johnsonii Lipoteichoic Acid: Enhancing Stability and Biofunctional Properties. ACS Appl. Bio Mater..

[74] Zhou Y., Xue K., Li Y., Huang H. (2026). Various Strategies for Intelligent Intestinal Protection, Adhesion, and Survival of Probiotics. Food Wellness.

[75] Shu D., Zhang T., Dong Y., Xu J., Yuan Y. (2026). Encapsulation of probiotics to improve their survival: A focus on *Bifidobacterium animalis* subsp. *lactis* BB-12. Food Chem. X.

[76] Ishihama H., Ishii K., Nagai S., Kakinuma H., Sasaki A., Yoshioka K., Kuramoto T., Shiono Y., Funao H., Isogai N. (2021). An antibacterial coated polymer prevents biofilm formation and implant-associated infection. Sci. Rep..

[77] Ramírez-Rodríguez G.B., Sabio L., Cerezo-Collado L., Garcés V., Domínguez-Vera J.M., Delgado-López J.M. (2025). Probiotic-Based Mineralized Living Materials to Produce Antimicrobial Yogurts. Adv. Healthc. Mater..

[78] Seredin P., Goloshchapov D., Litvinova T. (2024). Biomaterials and Agents: Pharmaceutical and Biomedical Applications in Dental Research. Pharmaceutics.

[79] Li L., Xu J., Ye C., Zhou Y., Yan F., Chen Z., Xiao Y. (2026). Biomaterials-based strategy for dental-oral tissue regeneration: Current clinical application, laboratory development, and future direction. Biomaterials.

[80] Bryers J.D., Ratner B.D. (2006). Biomaterials approaches to combating oral biofilms and dental disease. BMC Oral Health.

[81] Lou J., Xiang Z., Zhu X., Song J., Huang N., Li J., Jin G., Cui S., Xu P., Le X. (2025). Evaluating the therapeutic efficacy and safety of alginate-based dressings in burn wound and donor site wound management associated with burn surgery: A systematic review and meta-analysis of contemporary randomized controlled trials. BMC Surg..

[82] Diep E., Schiffman J.D. (2024). Living antimicrobial wound dressings: Using probiotic-loaded, alginate nanofibers for protection against methicillin-resistant Staphylococcus aureus. ACS Appl. Bio Mater..

[83] Soltani N., Abbasi S., Baghaeifar S., Taheri E., Farhoudi Sefidan Jadid M., Emami P., Abolhasani K., Aslanshirzadeh F. (2022). Antibacterial and antibiofilm activity of Lactobacillus strains secretome and extraction against *Escherichia coli* isolated from urinary tract infection. Biotechnol. Rep..

[84] Junkins E.N., McWhirter J.B., McCall L.I., Stevenson B.S. (2022). Environmental structure impacts microbial composition and secondary metabolism. ISME Commun..

[85] Wang Y., Pei Y., Wang X., Dai X., Zhu M. (2024). Antimicrobial metabolites produced by the plant growth-promoting rhizobacteria (PGPR): Bacillus and Pseudomonas. Adv. Agrochem.

